# SRF STEWART RHIND SCIENCE WRITING ESSAY: Why our current embryo experimentation laws are like a medical school toilet

**DOI:** 10.1530/RAF-21-0011

**Published:** 2021-05-13

**Authors:** Georgia May

**Affiliations:** 1 Department of Oncology and Metabolism, The University of Sheffield, Sheffield, UK

**Keywords:** embryo, reproductive medicine

## Abstract

This essay discusses the uncanny similarities between the toilets at the Leeds School of Medicine and our current regulations on embryo experimentation. Topics touched upon include the reasons behind the establishment of the 14-day rule on embryo culture, why there is a call for an extension to 28 days, and why this may be met with opposition. The author concludes that, much like the toilets at the Worsley building, our current legislation may no longer be fit for purpose and that re-opening the conversation about our 14-day rule is necessary in light of shifting scientific and societal opinion.

## Re-open the conversation about limiting human embryonic culture to 14 days in light of shifting scientific and societal opinion

In 1979 the Worsley building was completed, and since then has been a centre of learning to thousands of students at Leeds School of Medicine. When the Worsley building was built the majority of their medical student intake was male, and subsequently, the male toilets outnumber the female toilets by a ratio of around 80:20. This was not maliciously done, and perfectly suited the requirements of their student body at the time, it was probably never considered that the female medical students of the future might contribute more to their student cohort. Fast forward 40 years to the class of 2019, and female students now form the majority of the class intake (60%) (University of Leeds 2019). Thus the Worsley building now has an excess of male toilets, and lots of female medical students waiting in line. This change is not exclusive to the Leeds School of Medicine, and female medical students have exceeded their male counterparts since 1996 according to data presented by the Higher Education Funding Council for England (Moberly 2018). While this change is welcome as an indicator of progress in encouraging women to aspire to higher education, particularly in areas that have previously been male dominated, it means the Worsley building is no longer fit for purpose.

## How does this relate to human embryo research?

The Warnock Report was published in 1984 and provided the first guidance for the UK government on the ethics surrounding infertility treatments and research on human embryos (Warnock 1984). This advised the government on the regulation of these fields, ensuring that laws passed were well informed and ethically acceptable to the majority of the UK public while still considering the expert medical advice of the time.

A major point of debate was how long a human embryo could be kept alive outside the human body for research purposes. The committee were very aware that amongst the general public, and even amongst scientific experts, opinions varied significantly. At the time embryonic research was in its infancy, and a total ban on human embryo experimentation would have not only stood in the way of the significant improvements to IVF technologies seen over the last 30 years, but also wider medical advances in the understanding of early development and disease.

Because of the potential that embryonic culture held, the committee tentatively decided that 14 days would be the maximum limit that a human embryo could be kept in culture (Warnock 1984). This was predominantly due to human embryonic neuronal development beginning at approximately 17 days, which was determined to be the first point at which sentience or sensation of pain may occur. From here the committee conservatively took off a few days just to be certain (LaTourelle 2014). This debate was purely hypothetical at the time, as no human embryos had been cultured successfully far beyond a week, and focus was on choosing a number that was straightforward and easy to remember. Therefore this 14-day limit is relatively arbitrary and based on very little scientific reasoning, but has since been incorporated into law not only in the UK but in many countries around the world (Chan 2018). Subsequently, and perhaps incorrectly, it has become a ‘moral truth’ that human life becomes irretrievably valuable as soon as the calendar hits 2 weeks (Hyun *et al.* 2016).

Fast forward to the present and there have been increasing calls from the scientific community to re-open this debate with the view to extending the 14-day rule to 28 days (Appleby & Bredenoord 2018). Since the 80s there has been little change in the ethical arguments used for and against a longer embryo culture period, however, scientific advances and the gradual shift in society’s view of human embryos may provide up-to-date evidence to each side of the debate. And much like the toilets in the Worsley building, we must consider whether a previously suitable piece of legislation meets our current needs.

## Why might people support the extension of the 14-day rule?

A major consideration made when establishing the 14-day rule was the potential of a human embryo to experience sentience or pain, and how the establishment of neuronal development may mark the beginning of this (Warnock 1984). However, since 1984 there has been significant progress in developmental research which has indicated that the potential to feel pain may not begin until 7 weeks, and even this is a conservative estimate (Derbyshire 2006). Before 7 weeks there is no connection between the developing nerve endings and the area of the brain responsible for the sensation of pain (Andrews & Fitzgerald 1994). Without this anatomical connection, there is no capacity for pain, and therefore an increase in the limit to 28 days would not expose human embryos to any unnecessary discomfort.

Most people believe that human embryos hold a degree of value above their animal counterparts, and therefore awarding them extra rights seems self-evident. However, people rarely value a human embryo as equal to a pre-existing human, and more commonly both experts and the general public are aware that the benefit of research in alleviating existing suffering has potential to exceed the moral value of a human embryo. Increasingly with current medical and scientific advances, there is cause for extending the 14-day rule to promote better understanding of our early development and how stem cells work. This may provide answers to poorly understood conditions, such as neurodegenerative diseases and cancers, and help in the development of novel treatments, thus significantly benefiting the lives of many already living people. This reasoning is encouraged by recent research which has achieved what was previously considered a pipe dream - research groups have managed to culture human embryos up to the 14-day limit (Deglincerti *et al.* 2016, Shahbazi *et al.* 2016). This suggests we are closer than ever to be capable of exploring beyond 14 days and that longer embryo culture, with the associated medical potential, is tangible.

## Why might people oppose the extension of the 14-day rule?

It is important to consider the arguments against extending the 14-day rule, many of which were used at the time of its establishment against human embryo experimentation itself. While there is little scientific evidence to oppose the conservative extension of the 14-day rule, ethically this discussion becomes more complex. Moral objections are frequently difficult to unpick but are common and therefore cannot be discounted. This is a subjective discussion and often both experts and members of the general public will hold views on what ‘feels right’, intrinsic to their societal and personal morals and beliefs rather than scientific evidence.

Frequently these moral objections stem from religious beliefs, however, this is not always the case and questions of when life starts, and if an embryo has a right to life are the most common. People can feel unsure as to whether the potential benefits of research could ever outweigh the moral worth of an embryo, and there is a wide spectrum of answers to this question. It is also important to discuss when an embryo is considered a person, which can be any time between conception and birth and is often dependent on the particular religious beliefs held by an individual. Islam describes the point of ensoulment at the 134th day after conception, whereas the Catholic Church maintains that from the point of conception an embryo should be given full rights to life (Gowri 2013). While the concept that an embryo gradually achieves person-hood during its development has gained traction in recent years, it does not provide any conclusive support for a defined cut off. Because of this, erring on the side of caution could be considered the best option.

The ‘Slippery slope’ from extending the 14-day rule to more extreme, and perhaps less ethical, scientific practices in the future remains at the forefront of the opposing movement. This reasoning is commonly referenced in many scientific and ethical debates, and was used recently when debating the use of the mitochondrial donation for the treatment of hereditary mitochondrial disorders, suggesting that this may later lead to ‘designer babies’ in the future. To reference the Worsley Building again, one might consider the risk of a ‘slippery slope’ situation as if Leeds School of Medicine, after building additional female toilets, were then to remove all male toilets in favour of female. This would take positive change too far, and begin negatively impacting the male portion of their student body. While highly unlikely in this context, it remains a concern for many with regards to the 14-day rule and even the consideration of an extension could be considered proof of the existence of the ‘slippery slope’ to some.

## Looking to the future

Finding a balance between intricate ethical views and scientific evidence is complex, and inevitably falls to our Parliament. MPs, Lords, and organisations can initiate the writing of new bills such as one that could extend the 14-day rule. However, the public and scientific community can influence this through petitions, letters to members of Parliament, submission of evidence, and lobbying. This can work not only to draw attention to an issue and prompt the drafting of a new bill but can also influence how the bill is received in Parliament. Our Parliament needs public input and support to make sure that our legislation reflects our current societal and scientific views.

Much like the Leeds School of Medicine should take time to notice their changing student cohort, we have a responsibility to make sure that our parliament is in the best position possible to democratically represent our population. So this is a call to arms in the hope that not only may there be one less female medical student in need of a wee, but that by re-opening this conversation about our current laws on human embryo experimentation, we can ensure our legislation reflects the people of 2020.

## Declaration of interest

The author declares that there is no conflict of interest that could be perceived as prejudicing the impartiality of this review.
